# Factor H Is Bound by Outer Membrane-Displayed Carbohydrate Metabolism Enzymes of Extraintestinal Pathogenic *Escherichia coli* and Contributes to Opsonophagocytosis Resistance in Bacteria

**DOI:** 10.3389/fcimb.2020.592906

**Published:** 2021-01-25

**Authors:** Yu Sun, Bin Xu, Xiangkai Zhuge, Fang Tang, Xuhang Wang, Qianwen Gong, Rui Chen, Feng Xue, Jianjun Dai

**Affiliations:** ^1^ MOE Joint International Research Laboratory of Animal Health and Food Safety, College of Veterinary Medicine, Nanjing Agricultural University, Nanjing, China; ^2^ Key Laboratory of Animal Bacteriology, Ministry of Agriculture, Nanjing Agricultural University, Nanjing, China; ^3^ National Research Center of Veterinary Biologicals Engineering and Technology, Institute of Veterinary Medicine, Jiangsu Academy of Agricultural Sciences, Nanjing, China; ^4^ Department of Nutrition and Food Hygiene, School of Public Health, Nantong University, Nantong, China; ^5^ School of Life Science and Technology, China Pharmaceutical University, Nanjing, China

**Keywords:** complement system, factor H, extraintestinal pathogenic *Escherichia coli*, carbohydrate metabolism enzymes, opsonophagocytosis

## Abstract

Extraintestinal pathogenic *Escherichia coli* (ExPEC) causes bloodstream infections in humans and animals. Complement escape is a prerequisite for bacteria to survive in the bloodstream. Factor H (FH) is an important regulatory protein of the complement system. In this study, ExPEC was found to bind FH from serum. However, the mechanisms of ExPEC binding to FH and then resistance to complement-mediated attacks remain unclear. Here, a method that combined desthiobiotin pull-down and liquid chromatography-tandem mass spectrometry was used to identify the FH-binding membrane proteins of ExPEC. Seven identified proteins, which all were carbohydrate metabolic enzymes (CMEs), including acetate kinase, fructose-bisphosphate aldolase, fumarate reductase flavoprotein subunit, L-lactate dehydrogenase, dihydrolipoamide dehydrogenase, phosphoenolpyruvate synthase, and pyruvate dehydrogenase, were verified to recruit FH from serum using GST pull-down and ELISA plate binding assay. The ELISA plate binding assay determined that these seven proteins bind to FH in a dose-dependent manner. Magnetic beads coupled with any one of seven proteins significantly reduced the FH recruitment of ExPEC (*p* < 0.05) Subsequently, immunofluorescence, colony blotting, and Western blotting targeting outer membrane proteins determined that these seven CMEs were located on the outer membrane of ExPEC. Furthermore, the FH recruitment levels and C3b deposition levels on bacteria were significantly increased and decreased in an FH-concentration-dependent manner, respectively (*p* < 0.05). The FH recruitment significantly enhanced the ability of ExPEC to resist the opsonophagocytosis of human macrophage THP-1 in an FH-concentration-dependent manner (*p* < 0.05), which revealed a new mechanism for ExPEC to escape complement-mediated killing. The identification of novel outer membrane-displayed CMEs which played a role in the FH recruitment contributes to the elucidation of the molecular mechanism of ExPEC pathogenicity.

## Introduction

Extraintestinal pathogenic *Escherichia coli* (ExPEC) is an important pathogen that causes extraintestinal infections, including urinary tract infection, bloodstream infection, and meningitis in humans and animals (Russo and Johnson, 2000; Zhuge et al., 2018). Studies have shown that a high level of ExPEC proliferation in the blood is the prerequisite for bloodstream infections (Xie et al., 2004). In addition, avoiding the defense of host immune system facilitates ExPEC infections. However, the mechanism by which ExPEC escapes the immune system in bloodstream infections remains unclear.

The complement system is an important component of the serum, serving as the critical line of the immune defense system to control bacterial infections (Dalia and Weiser, 2011). There are three activation pathways in the complement system, namely, the classical pathway (CP) that is mediated by antibodies bound to the surface of the pathogen, the lectin pathway (LP) that is mediated by the recognition of carbohydrates on the surface of the pathogen, and the alternative pathway (AP) that is mediated by the spontaneous cleavage of the C3 component (Harboe and Mollnes, 2008). Regardless of complement activation through the CP, LP, or AP, the C3b formation is an essential response step. When C3b is deposited on the surface of pathogens, it leads to the formation of membrane attack complexes (MACs) that causes the lysis of pathogens (Jozsi et al., 2015). Moreover, the deposited C3b opsonizes pathogens and promotes their recognition and phagocytosis by phagocytes such as macrophages (Parente et al., 2017). The C3b deposition plays a key role of in complement activation. To avoid destruction of host cells mediated by complement, the complement system has to be strictly regulated. Factor H (FH), one of the key negative regulators of complement activation, inhibits the formation of C3 convertase and promotes its degradation, thereby reducing the conversion of C3 to C3b. In addition, FH assists Factor I cleaving C3b into inactive C3b (Cabezas et al., 2018).

There are many pathogens surviving in serum despite exposure to the complement system. Bacteria have adapted mechanisms to evade the complement-mediated attack, including recruiting FH onto their surfaces (Lambris et al., 2008). For Gram-negative bacteria, *Neisseria meningitidis* expresses FH-binding protein (fHbp) on their surface to recruit FH, resulting in the decreased C3b deposition, which improves the survival ability of bacteria in serum (Granoff et al., 2009; Schneider et al., 2009). *Pseudomonas aeruginosa* utilizes elongation factor Tuf (EF-Tu) on bacterial surface to bind FH, resulting in the degradation of C3b and improvement of the survival ability of bacteria in human plasma (Kunert et al., 2007). For Gram-positive bacteria, *Streptococcus suis* employs the surface-located fHbp to interact with FH, which inhibits C3b deposition and inhibits phagocytic activities (Pian et al., 2012). *Streptococcus pneumoniae* uses Pneumococcal surface protein C to bind to FH, reducing the C3b deposition and opsonization, which enhances the serum resistance of bacteria (van der Maten et al., 2016). Many FH-binding proteins are outer membrane proteins such as fHbp. Furthermore, some FH-binding proteins are moonlighting proteins such as EF-Tu.

ExPEC possesses numerous membrane proteins. Unfortunately, investigations on the FH-binding protein in ExPEC are limited. Important potential FH-binding proteins have yet to be identified. In this study, the membrane proteins of ExPEC with FH-binding function were screened. The FH-binding activity and the outer membrane location of seven identified carbohydrate metabolic enzymes (CMEs) of ExPEC were explored. In Gram-positive bacteria, the acquisition of FH on bacteria causes significant decrease in C3b deposition, resulting in the impairment of opsonophagocytosis (Schindler et al., 2012; van der Maten et al., 2018). However, whether FH binding affects C3b deposition and opsonization of ExPEC remains unclear. This study investigated these effects to assist in the elucidation of the complement evasion mechanism in ExPEC bloodstream infections.

## Methods and Materials

### Strains, Plasmids, Serum, and Cell Line

The bacterial strains and plasmids used in this study are presented in **Table 1**. The ExPEC strain RS218 was isolated from the cerebrospinal fluid in neonates with meningitis (Mittal and Prasadarao, 2011). All tests involving live pathogenic *E. coli* were done under in the level 2 biosafety laboratory (BSL) facility. The *E. coli* K-12 MG1655 is a non-pathogenic strain (Stromberg et al., 2018). *E. coli* strains DH5α (Vazyme, Cat# C502-02) and BL21 (Vazyme, Cat# C504-02) were employed in plasmid cloning and expression of the recombinant protein, respectively. The vectors pET-28a (Novagen, Cat# 69864-3) and pGEX-4T1 (Amersham, Cat# 27458001) were used for the construction of His-tag and GST-tag recombinant expression plasmids, respectively. Human serum (Sigma-Aldrich, Cat# H4522) was purchased from Sigma-Aldrich, which was obtained from human male AB plasma without complement inactivation. THP-1 cells (human macrophage cell line, CLS Cat# 300356/p804_THP-1, RRID: CVCL_0006) were cultured in RPMI 1640 media (Gibco, Cat# 72400047) with 10% fetal bovine serum (FBS, Gibco, Cat# 10100139C) at 37°C and 5% CO_2_ atmosphere. To induce a macrophage-like phenotype, phorbol 12-myristate 13-acetate (PMA, Sigma-Aldrich, Cat# P8139) was added at a concentration of 50 ng/ml and maintained for another 24 h (Skjesol et al., 2019).

**Table 1:** Bacterial strains and plasmids used in this study.

Names	Notable characteristic(s)^a^	Source or reference
Bacteria		
DH5α	Host for plasmid cloning	Purchased from Vazyme
BL21	Host for recombinant protein expression	Purchased from Vazyme
RS218	Virulent strain of ExPEC from the cerebrospinal fluid in neonates with meningitis	Mittal and Prasadarao, 2011
MG1655	Non-pathogenic *E. coli* K-12	Stromberg et al., 2018
Plasmids		
pET-28a	Prokaryotic recombinant protein expression plasmid, Kan ^r^	Purchased from Novagen
pET-28a:: *ackA*	pET-28a containing *ackA* in the *BamHI*-*SalI* restriction sites, Kan ^r^	This study
pET-28a:: *fbaA*	pET-28a containing *fbaA* in the *EcoRI*-*XhoI* restriction sites, Kan ^r^	This study
pET-28a:: *frdA*	pET-28a containing *frdA* in the *EcoRI*-*XhoI* restriction sites, Kan ^r^	This study
pET-28a:: *ldh*	pET-28a containing *ldh* in the *BamHI*-*SalI* restriction sites, Kan ^r^	This study
pET-28a:: *lpdA*	pET-28a containing *lpdA* in the *BamHI*-*SalI* restriction sites, Kan ^r^	This study
pET-28a:: *pdh*	pET-28a containing *pdh* in the *SalI*-*XhoI* restriction sites, Kan ^r^	This study
pET-28a:: *pgk*	pET-28a containing *pgk* in the *BamHI*-*SalI* restriction sites, Kan ^r^	This study
pET-28a:: *ppsA*	pET-28a containing *ppsA* in the *BamHI*-*SalI* restriction sites, Kan ^r^	This study
pET-28a:: *ompA*	pET-28a containing *ompA* in the *EcoRI*-*XhoI* restriction sites, Kan ^r^	This study
pGEX-4T1	Prokaryotic recombinant protein expression plasmid, Amp ^r^	Purchased from Amersham,
pGEX-4T1:: *ackA*	pGEX-4T1 containing *ackA* in the *BamHI*-*SalI* restriction sites, Amp ^r^	This study
pGEX-4T1:: *fbaA*	pGEX-4T1 containing *fbaA* in the *EcoRI*-*XhoI* restriction sites, Amp ^r^	This study
pGEX-4T1:: *frdA*	pGEX-4T1 containing *frdA* in the *EcoRI*-*XhoI* restriction sites, Amp ^r^	This study
pGEX-4T1:: *ldh*	pGEX-4T1 containing *ldh* in the *BamHI*-*SalI* restriction sites, Amp ^r^	This study
pGEX-4T1:: *lpdA*	pGEX-4T1 containing *lpdA* in the *BamHI*-*SalI* restriction sites, Amp ^r^	This study
pGEX-4T1:: *pdh*	pGEX-4T1 containing *pdh* in the *BamH* I-*Sal* I restriction sites, Amp ^r^	This study
pGEX-4T1:: *pgk*	pGEX-4T1 containing *pgk* in the *BamHI*-*SalI* restriction sites, Amp ^r^	This study
pGEX-4T1:: *ppsA*	pGEX-4T1 containing *ppsA* in the *BamHI*-*SalI* restriction sites, Amp ^r^	This study

a Kan ^r^, kanamycin resistance; Amp ^r^, Ampicillin resistance.

### Bacterial FH-Binding Assay

ExPEC strain RS218 was cultured in LB medium up to the mid-log phase at 37°C. Bacteria were harvested by centrifugation and washed with PBS. Bacteria were adjusted to a density of 1.0 × 10^7^ CFUs per assay. Then, the bacteria were incubated with 10% human serum solution in PBS at 37°C for 1 h. Bacteria incubated with 2% BSA were used as a negative control. After washing five times with PBS, the bacteria and the last washing buffer were subjected to SDS-PAGE and then transferred onto a polyvinylidene difluoride (PVDF) membrane. Human serum was used as positive control (Kunert et al., 2007). After blocking with PBST (0.05% Tween-20 in PBS) containing 5% non-fat milk at 4°C overnight, the PVDF membrane was incubated with mouse anti-human FH monoclonal antibody (Abcam Cat# ab118820, RRID: AB_10899656) for 2 h. After three washes with PBST, the PVDF membrane was incubated with HRP-conjugated rabbit anti-mouse IgG antibody (Abcam, Cat# ab7075, RRID: AB_955429) for 1 h. Then the PVDF membrane was washed with PBST for another three times. Western ECL Subs (Bio-Rad, Cat# 1705070) and Bio-Rad ChemiDoc™ Touch Imaging System (Bio-rad) were employed for immunoblotting detection.

The whole-bacteria ELISA assay was performed as previously described (Biedzka-Sarek et al., 2008). Bacteria were incubated with 10% human serum or 2% BSA at 37°C for 1 h, and then washed five times with PBS. Bacteria (1.0 × 10^6^ CFUs) were resuspended in carbonate buffer and immobilized in a 96-well ELISA plate overnight at 4°C. After washing with PBST, the wells were blocked with 0.5% BSA. The plates were washed thrice with PBST. Mouse anti-human FH monoclonal antibody was then added. Following incubation, the wells were washed. The HRP-conjugated rabbit anti-mouse IgG antibody was added and incubated for another 1 h, and then the wells were washed four times with PBST. TMB substrate solution (200 μl, TIANGEN, Cat# PA107) was added and incubated at 37°C for 10 min. After adding 50 μl of 2 M H_2_SO_4_, the OD_450_ was measured using a Tecan Spark Reader. The ELISA assay was performed thrice.

### Extraction of ExPEC Membrane Proteins

ExPEC membrane proteins were extracted as previously described with some modifications (Bordier, 1981). Briefly, ExPEC strain RS218 was cultured up to the log phase. Bacteria were harvested and washed twice with PBS. Then the bacterial pellet was resuspended in PBS with 1.0% Triton X-114 and incubated at 4°C for 4 h. The insoluble material was discarded by centrifugation at 10,000*g* for 10 min. The supernatant was subjected to phase separation at 37°C for 15 min and centrifuged at 25°C for 15 min. The detergent phase was the ExPEC membrane proteins solution.

### EZ-Link Desthiobiotinylation and Pull-Down Assay

According to the instruction of the EZ-Link Desthiobiotinylation and Pull-Down Kit (Thermo Fisher Scientific, Cat# 16138), purified human FH (Merck, Cat# 341274) was desthiobiotin-labeled using 15-fold molar excess EZ-Link sulfo-NHS-LC-desthiobiotin solution. Excess desthiobiotin reagents were removed using a desalting column (Thermo Fisher Scientific, Cat# 89891). Then, the desthiobiotin-FH samples were incubated with streptavidin agarose resins at 4°C for 2 h. Desthiobiotin-glycine was used as a control. The agarose resin was washed thrice with PBS and incubated with the ExPEC membrane protein solution at 4°C for 4 h. After thorough washing, the bound membrane proteins were eluted with the biotin elution buffer.

### Liquid Chromatography-Tandem Mass Spectrometry and Database Queries

The FH-binding protein solutions were sent to Shanghai Applied Protein Technology Co., Ltd., for digestion. The peptides mixtures were separated by reversed-phase liquid chromatography for 60 min using the Easy nLC1000 System (ThermoFisher Scientific) and analyzed by tandem mass spectrometry (MS) using a Q-Exactive mass spectrometer (ThermoFisher Scientific). MS data were acquired using Q-Exactive software. The most abundant precursor ions from the survey scan (300–1,800 m/z) were collected as MS data using the data-dependent top 20 method.

The MaxQuant version 1.5.5.1 (RRID : SCR_014485) were used to analyze MS data. The MS data were queried against *E. coli* RS218 (GenBank Accession No. CP007149.1). The settings used for the MaxQuant analysis were as follows: the enzyme was trypsin, two missed cleavages were allowed, the variable modifications were methionine oxidation, and the carbamidomethylation on cysteine was set as a fixed modification. A mass tolerance of 20 ppm was used for peptide, and a tolerance of 0.1 Da was used for fragment. The cutoff of global false discovery rate (FDR) for peptide and protein identification was set to 0.01. The intensity-based absolute-protein quantification (iBAQ) was true.

### Expression and Purification of Recombinant Proteins, and Preparation of Antibodies

To express His-tag recombinant outer membrane protein (OmpA) and seven CMEs, namely, acetate kinase (AckA), fructose-bisphosphate aldolase (FbaA), fumarate reductase flavoprotein subunit (FrdA), L-lactate dehydrogenase (LDH), dihydrolipoamide dehydrogenase (LpdA), pyruvate dehydrogenase (Pdh), phosphoglycerate kinase (Pgk), and phosphoenolpyruvate synthase (PpsA), the ExPEC strain RS218 genomic DNA was used as the template for PCR using the primers listed in **Table 2**. Amplicons were cloned into the expression vector pET-28a using the ClonExpress II One Step Cloning Kit (Vazyme, Cat# C112-01) according to manufacturer instructions. Recombinant plasmids were transformed into *E. coli* BL21. When bacteria had grown to an OD_600_ of 0.6, 1 mM isopropyl-β-d-thiogalactopyranoside (IPTG) was added to induce protein expression at 16°C for 8 h. Protein purification was performed by Ni-chelating chromatography (GE Healthcare, Cat# 17524701) according to the instruction manual.

**Table 2:** Primers used in this study.

Names	Oligonucleotide sequence (5’–3’)^a^	Products
AckA-His-F	ATGGGTCGCGGATCCATGTCGAGTAAGTTAGTA	*ackA*
AckA-His-R	GCAAGCTTGTCGACGTCAGGCAGTCAGGCGGC	
AckA-GST-F	GTTCCGCGTGGATCCATGTCGAGTAAGTTAGTA	
AckA-GST-R	CCGCTCGAGTCGACCTCAGGCAGTCAGGCGGC	
FbaA-His-F	CGCGGATCCGAATTCATGTCTAAGATTTTTGATT	*fbaA*
FbaA-His-R	GTGGTGGTGCTCGAGTTACAGAACGTCGATCGC	
FbaA-GST-F	GGATCCCCGGAATTCATGTCTAAGATTTTTGATT	
FbaA-GST-R	ATGCGGCCGCTCGAGTTACAGAACGTCGATCGC	
FrdA-His-F	CGCGGATCCGAATTCGTGCAAACCTTTCAAGCC	*frda*
Frda-His-R	GTGGTGGTGCTCGAGTCAGCCATTCGCCTTCTCC	
FrdA-GST-F	TGGATCCCCGGAATTCGTGCAAACCTTTCAAGCC	
Frda-GST-R	ATGCGGCCGCTCGAGTCAGCCATTCGCCTTCTCC	
LDH-His-F	ATGGGTCGCGGATCCATGATTATTTCCGCAGCCAG	*ldh*
LDH-His-R	GCAAGCTTGTCGACGTTAAGCTGCATTCCCTTTCG	
LDH-GST-F	GTTCCGCGTGGATCCATGATTATTTCCGCAGCCAG	
LDH-GST-R	CCGCTCGAGTCGACCTTAAGCTGCATTCCCTTTCG	
LpdA-His-F	ATGGGTCGCGGATCCATGAGTACTGAAATCAAAA	*lpdA*
LpdA-His-R	GCAAGCTTGTCGACGTTACTTCTTCTTCGCTTTCG	
LpdA-GST-F	GTTCCGCGTGGATCCATGAGTACTGAAATCAAAA	
LpdA-GST-R	CCGCTCGAGTCGACCTTACTTCTTCTTCGCTTTCG	
Pdh-His-F	TTCGAGCTCCGTCGAATGTCAGAACGTTTCCC	*pdh*
Pdh-His-R	GTGGTGGTGCTCGAGTTACGCCAGACGCGGG	
Pdh-GST-F	GTTCCGCGTGGATCCATGTCAGAACGTTTCCC	
Pdh-GST-R	CCGCTCGAGTCGACCTTACGCCAGACGCGGG	
Pgk-His-F	ATGGGTCGCGGATCCATGTCTGTAATTAAGATGAC	*pgk*
Pgk-His-R	GCAAGCTTGTCGACGTTACTTCTTAGCGCGCTCTT	
Pgk-GST-F	GTTCCGCGTGGATCCATGTCTGTAATTAAGATGAC	
Pgk-GST-R	CCGCTCGAGTCGACCTTACTTCTTAGCGCGCTCTT	
PpsA-His-F	ATGGGTCGCGGATCCATGTCCAACAATGGCTCGTC	*ppsA*
PpsA-His-R	GCAAGCTTGTCGACGTTATTTCTTCAGTTCAGCCA	
PpsA-GST-F	GTTCCGCGTGGATCCATGTCCAACAATGGCTCGTC	
PpsA-GST-R	CCGCTCGAGTCGACCTTATTTCTTCAGTTCAGCCA	
OmpA-His-F	CGCGGATCCGAATTCATGGCTCCGAAAGATAACAC	*ompA*
OmpA-His-R	GTGGTGGTGCTCGAGTTAAGCCTGCGGCTGAGTTA	

a Underlined sequences of primers correspond to restriction enzyme recognition sites. GTCGAC, SalI; GGATCC, BamHI; GAATTC, EcoRI; CTCGAG, XhoI.

Antibodies preparation was completed by Shanghai Willget Biotechnology Co., Ltd. The peptides used for immunization were designed and synthesized based on the amino acid sequence of CMEs and OmpA. Rabbits were immunized with peptides to obtain serum, and then specific antibodies were obtained by affinity purification of the antigen. **Supplementary Table S1** lists peptide sequences used for antibodies preparation.

### ELISA Plate Binding and GST Pull-Down Assays

For the ELISA plate binding assay, the 96-well ELISA plate was immobilized overnight at 4°C with 100 μl of His-tag recombinant proteins AckA, FbaA, FrdA, LDH, LpdA, Pdh, Pgk, PpsA, and BSA (0.1, 1.0, 10, and 20 μg/ml). After blocking, a 10% human serum solution was added to the plate and incubated at 37°C for 1 h. The remaining incubations were conducted using the instructions provided in the whole-bacteria ELISA assay (Kunert et al., 2007). Assays were repeated as three independent experiments.

GST pull-down assay was performed according to a previous study with some modifications (Alitalo et al., 2004). These eight CME-encoding genes were subcloned into the pGEX-4T-1 expression vector, using the ClonExpress II One Step Cloning Kit. The primers used in this assay are listed in **Table 2**. *E. coli* BL21 harboring the empty plasmid pGEX-4T-1 that expressed the GST tag was used as a negative control. After induction with IPTG, bacteria were harvested and subjected to ultrasonication. After centrifugation, the soluble GST tag or GST-fusion protein was incubated with glutathione magnetic beads (Enriching Biotechnology, Cat# p32-010) with gentle rotation at 4°C for 2 h. The magnetic beads were washed five times with cold PBST and then incubated with 10% human serum on a gentle rotation at 37°C for 1 h. Then the magnetic beads were washed five times with PBST. The bound proteins were subjected to SDS-PAGE and Western blotting and analyzed using mouse anti-GST monoclonal antibody (Abcam, Cat# ab34589, RRID: AB_732632) and mouse anti-FH monoclonal antibody.

### FH Binding Inhibition Assay of Magnetic Beads Coupled With Recombinant Proteins

GST-fusion recombinant proteins AckA, FbaA, FrdA, LDH, LpdA, Pdh, Pgk, and PpsA, and GST tag protein (2 μg) were incubated with glutathione magnetic beads at 4°C for 2 h, respectively. The unbound proteins were washed off with PBST. Then magnetic beads coupled with GST recombinant proteins and GST tag were co-incubated with ExPEC strain RS218 (1.0 × 10^7^ CFUs) in 10% human serum at 37°C for 1 h, respectively. The magnetic beads were separated by a magnet. After centrifugation, the bacteria were washed with PBS for five times. Then 1.0 × 10^6^ CFUs cells were immobilized overnight at 4°C in 96-well ELISA plates. The anti-FH monoclonal antibody and HRP-conjugated rabbit anti-mouse IgG antibody were used to detect FH on ExPEC. This assay was performed thrice.

### Localization of CMEs on ExPEC Membrane

To investigate the possible exposure of these CMEs on the outer membrane of ExPEC, suspension immunofluorescence assays were conducted as described elsewhere (Qi et al., 2018). Freshly grown ExPEC strain RS218 was harvested in 1.5 ml-Eppendorf tubes. After fixing with formaldehyde for 30 min and blocking with BSA for 1 h, the following mixtures of antibodies or antiserums were added: mouse anti-RS218 anti-serum and rabbit anti-RS218 anti-serum or rabbit pre-immune serum (PIS), mouse anti-RS218 anti-serum and seven different rabbit anti-CME antibodies, mouse anti-RS218 anti-serum and rabbit anti-OmpA antibody or rabbit anti-LexA antibody (Abcam, Cat# ab174384), rabbit anti-RS218 anti-serum and mouse PIS. After incubation at 37°C for 1 h, the bacteria were washed and the mixture of FITC-labeled anti-mouse IgG (Abcam, Cat# ab6785, RRID: AB_955241) and TRITC-labeled anti-rabbit IgG (Abcam, Cat# ab6718, RRID: AB_955551) were added, and incubated at 37°C for 30 min. The bacteria were washed and resuspended in PBS and the immunofluorescence was observed under a fluorescence microscope (Zeiss) at 100× magnification.

To further detect the subcellular localization of these CMEs, colony blotting was performed as earlier described (Gründel et al., 2015; Qi et al., 2018). ExPEC strain RS218 was inoculated onto the LB agar and incubated overnight. Bacterial colonies were covered with nitrocellulose filter (NC) membranes for 5 min. The NC membranes were dried for 10 min at room temperature, rinsed with PBST, and blocked with 5% non-fat milk. Then, the NC membranes were hybridized with rabbit anti-RS218 anti-serum, rabbit anti-CME antibodies, rabbit anti-OmpA antibody, rabbit anti-LexA antibody, or rabbit PIS. Detection was carried out using HRP-conjugated anti-rabbit IgG (Abcam, Cat# ab6721, RRID: AB_955447).

The surface proteins of ExPEC were labeled with EZ-Link sulfo-NHS-LC-desthiobiotin as previously described with some modifications (Gibson et al., 2010; Cho and Roche, 2019). The fresh ExPEC RS218 were collected and washed with PBS. Bacteria (1.0 × 10^9^ CFUs) were resuspended in 1 ml PBS containing 1 mg EZ-Link sulfo-NHS-LC-desthiobiotin. The biotinylation reaction was carried out on ice for 30 min. Excessive desthiobiotin was quenched by washing bacteria in 500 mM glycine-PBS 3 times. As described above, the membrane proteins were extracted using Triton X-114. Then the labeled outer membrane proteins were isolated from membrane proteins using streptavidin agarose resins. Proteins were subjected to SDS-PAGE and transferred onto a PVDF membrane. The rabbit anti-OmpA antibody, rabbit anti-CME antibodies, or the rabbit anti-LexA antibody and HRP-conjugated anti-rabbit IgG were used for immunoblotting detections.

### FH Recruitment and C3b Deposition Assays

The FH-depleted human serum was prepared as described with modifications (Potempa et al., 2008). Human serum was incubated with Protein A/G magnetic agarose beads (Thermo Scientific, Cat# 78609) coupled with FH antibody according to the manufacturer’s instruction. The FH-depleted serum was analyzed by Western blotting and preserved in -80°C. The C3b deposition assays were carried out according to previous research with some modifications (Ma et al., 2012; van der Maten et al., 2018). Fresh ExPEC RS218 cells were centrifuged and washed thrice with PBS. The bacteria (1.0 × 10^7^ CFUs) were incubated with the purified human FH protein (1.0, 5.0, 10, or 20 μg/ml) or BSA in 1 ml PBS at 37°C for 30 min, respectively. After washing, the bacteria were treated with 500 μl 10% FH-depleted human serum containing 5 mM ethylene glycol tetra acetic acid (EGTA) and 5 mM MgCl_2_ and incubated at 37°C for 30 min. To stop complement activation, ethylenediaminetetraacetic acid (EDTA, 10 mM) was added to the reaction. The bacteria were washed with PBS. Then 1.0 × 10^6^ CFUs were immobilized to the 96-well plate for ELISA assay. The FH monoclonal antibody or the complement C3b monoclonal antibody (Thermo Fisher Scientific Cat# MA1-70053, RRID: AB_1073823) and HRP-conjugated anti-mouse IgG were used to detect FH recruitment and C3b deposition. Tests were performed thrice.

### Opsonophagocytosis Assay

THP-1 cells were seeded onto the 24-well plates at a density of 2.0 × 10^5^ cells/well, and each well was supplemented with 50 ng/ml PMA for 24 h. ExPEC strain RS218 was cultured to mid-log phase. The bacteria were collected and washed twice with PBS. The bacteria were opsonized as described in the *FH Recruitment and C3b Deposition Assays*. The bacteria were washed thrice with PBS and resuspended in FBS-free RPMI 1640 media. THP-1 cells were infected with bacteria at an MOI of 10. To exclude dead bacteria caused by the bactericidal effect of human serum, the same number of bacteria were used for plate counting. Gentamicin was added to kill extracellular bacteria after the THP-1 cells were infected for 1 h. After another 1 h, THP-1 cells were washed with PBS and lysed with 0.5% Triton X-100. The phagocytosed bacteria in THP-1 cells were harvested and counted by plate counting. The phagocytic rates were calculated as the ratio of the number of bacteria recovered from THP-1 cells to the number of those recovered from human serum. Experiments were repeated thrice.

### Statistical Analysis

GraphPad Prism version 8.0.1 (RRID: SCR_002798) was used to analyze data. The data were expressed as the standard error of the mean (SEM) or mean ± standard deviation (SD) values. The unpaired *t* test was used for pairwise comparisons and *p* < 0.05 was considered significant.

## Results

### ExPEC Bound to FH

**Figure 1:**
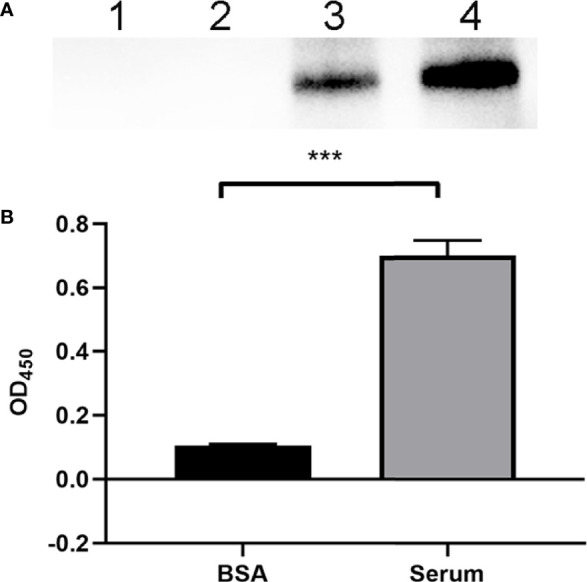
Identification of the FH-binding ability of ExPEC. **(A)** Western blotting to detect the ability of ExPEC to bind FH. ExPEC strain RS218 was incubated with 10% human serum or BSA. The bacteria, the last washing buffer, and human serum were analyzed by Western blotting. Lane 1, the bacteria after incubated with BSA (negative control); lane 2, the last washing buffer; lane 3, the bacteria after incubated with human serum; and lane 4, the human serum. **(B)** Whole-bacteria ELISA assay to detect the binding of FH after ExPEC incubated with human serum. ExPEC incubated with BSA acted as the negative control. Data are expressed as the mean ± SEM of three independent experiments. Statistical analysis was performed using the unpaired *t* test (***p < 0.001).

To evaluate the FH-binding ability of ExPEC, Western blotting was performed to assess the FH recruitment after the incubation of ExPEC with human serum. **Figure 1A** shows that a band of 150 kDa relative to the size of FH protein was detected in the bacteria that were incubated with human serum, whereas no band was observed when the bacteria were incubated with BSA. No band was detected in the last washing buffer, indicating that any unbound FH proteins were effectively washed off. The FH-binding ability of ExPEC was also detected by whole-bacteria ELISA assay. The results showed that the OD_450_ value of the control (BSA) group was 0.1047 ± 0.0044, whereas that of the human serum-treated group was 0.7001 ± 0.0477. The OD_450_ value of the serum-treated group was significantly higher than that of the BSA group (*p* < 0.5, **Figure 1B**). These results indicated that ExPEC possessed the ability to bind FH in human serum.

### The Interaction of CMEs With FH

Using a combination of the desthiobiotin pull-down assay and LC-MS/MS analysis, potential FH-binding proteins were identified (**Supplementary Table S2**). Subsequently, we selected eight identified CMEs, namely, AckA, FbaA, FrdA, LDH, LpdA, Pdh, Pgk, and PpsA for further analysis. The LC-MS/MS data of these above eight CMEs and the results of bioinformatics analysis are presented in **Table 3**.

**Table 3:** Identification of CMEs by LC-MS/MS and bioinformatics analysis.

Identified proteins	Accession No.	TheoreticalMW	Sequence coverage (%)	iBAQ	Prediction of signal peptide^a^	Prediction of transmembrane helices^b^
AckA	AJM74481.1	43.29	8	904,750	No	No
FbaA	AJM75116.1	39.205	14.8	9,455,300	No	No
FrdA	AJM76424.1	65.989	42	48,106,000	No	No
LDH	AJM75807.1	42.77	3.5	589,160	No	No
LpdA	AJM72343.1	50.688	5.3	548,190	No	No
Pdh	AJM72341.1	99.667	6.2	396,470	No	No
Pgk	AJM75117.1	41.118	24	8,085,400	No	No
PpsA	AJM73840.1	87.451	3.5	1,475,500	No	No

a The signal peptide was predicted by SignalP-5.0 Server (RRID : SCR_015644).

b The transmembrane helices were predicted by TMHMM Server version 2.0 (RRID : SCR_014935).

**Figure 2:**
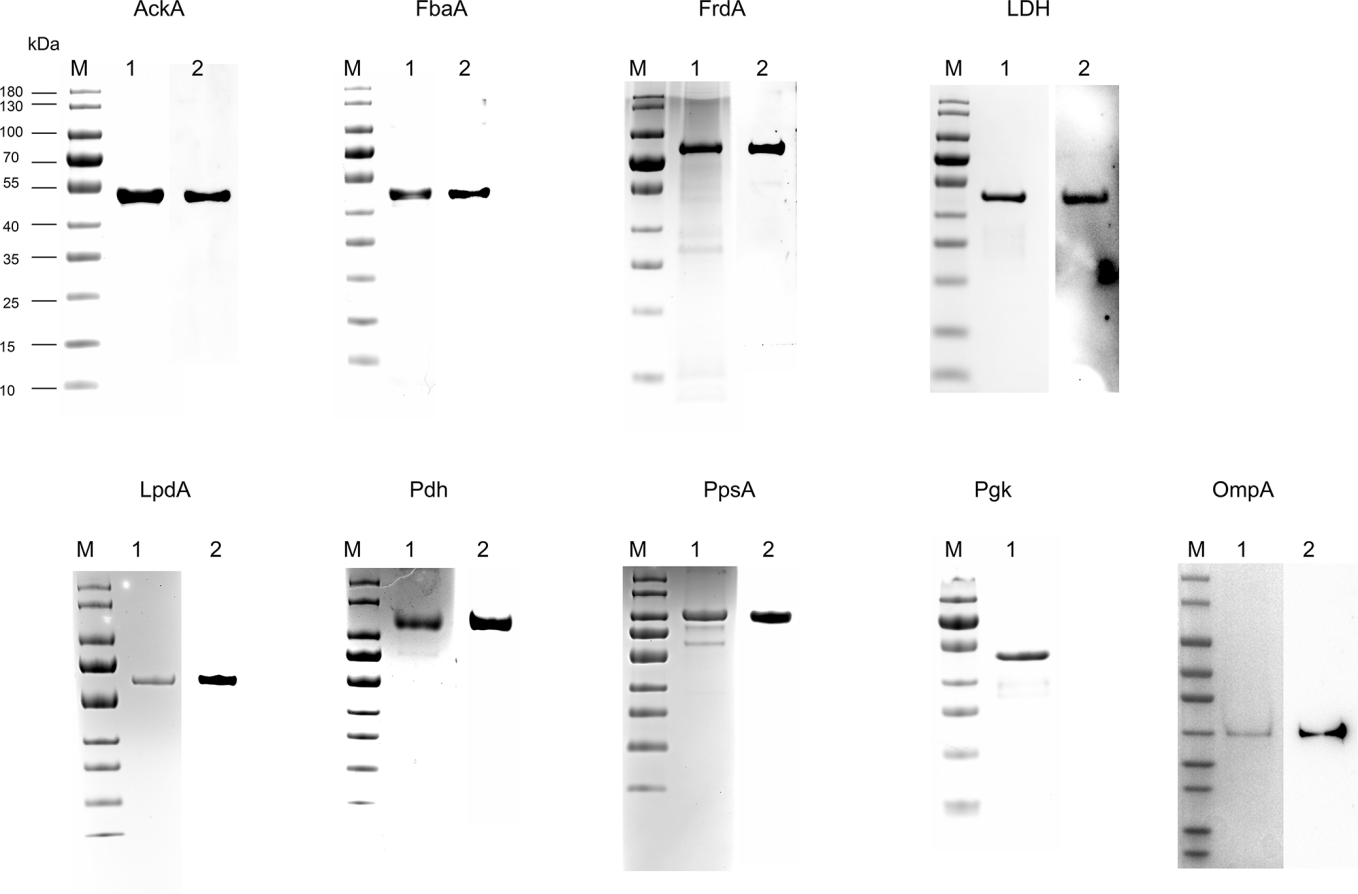
Expression of recombinant proteins and preparation of corresponding antibodies. His-tag recombinant protein AckA, FbaA, FrdA, LDH, LpdA, Pdh, PpsA, Pgk, and OmpA, were analyzed by SDS-PAGE and stained with Coomassie G-250 (lane 1 in each panel). The recombinant proteins (except Pgk) were hybridized to their specific polyclonal antibodies and HRP-conjugated anti-rabbit IgG (lane 2 in each panel).

**Figure 3:**
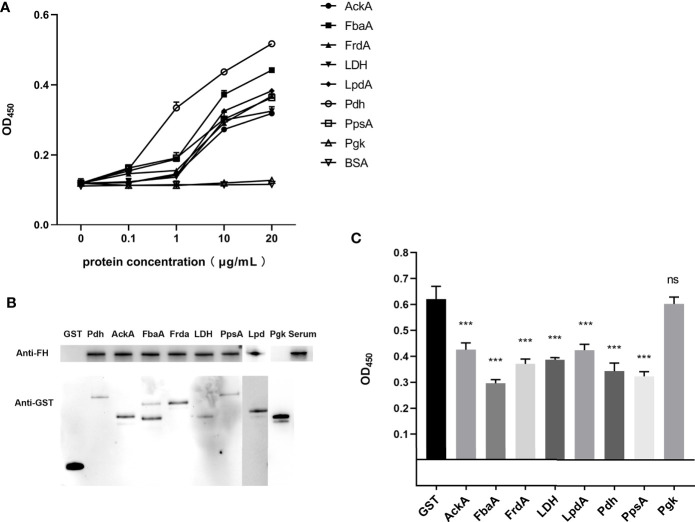
Identification of the FH-binding ability of CMEs in ExPEC. **(A)** ELISA plates were coated with the indicated concentrations of His-tag recombinant proteins or BSA, and then incubated with human serum. The bound FH was detected. Data are expressed as the mean ± SD of three independent experiments. The statistical significance was determined using the unpaired *t* test (***p < 0.001). **(B)** GST pull-down assay was carried out to confirm the interaction between FH and CMEs. The upper part of the panel B shows the FH-binding ability of the GST-fusion protein, and the lower part of panel B shows the results of the detection of the GST-fusion proteins. **(C)** FH binding inhibition assay of magnetic beads coupled with recombinant proteins. Magnetic beads coupled with GST-fusion proteins or GST protein were co-incubated with ExPEC in 10% human serum solution. The ExPEC FH-binding levels were detected by ELISA. The statistical significance was determined using the unpaired *t* test (***p < 0.001). Data are expressed as the mean ± SEM of three independent experiments.

First, the results of SDS-PAGE of His-tag recombinant proteins AckA, FbaA, FrdA, LDH, LpdA, Pdh, PpsA, and Pgk are shown in **Figure 2** (lane 1). To determine the FH-binding ability of these eight CMEs, increasing concentrations of recombinant proteins were immobilized on the ELISA plate, and the OD_450_ levels after interaction with human serum were determined. The results indicated that recombinant proteins AckA, FbaA, FrdA, LDH, LpdA, Pdh, and PpsA interacted with FH in a dose-dependent manner (**Figure 3A**). Under same conditions, Pgk and negative control BSA failed to interact with FH (**Figure 3A**).

For the GST pull-down assay, GST-fusion proteins were used as bait proteins to pull-down FH in human serum. **Figure 3B** confirms the expression of these fusion proteins by detecting the GST tag in Western blotting. Bands of FH showed that the GST-fusion proteins AckA, FbaA, FrdA, LDH, LpdA, Pdh, and PpsA could pull-down FH in human serum, indicating the interaction between these seven CMEs and FH. The GST-fusion protein of Pgk and GST tag did not interact with FH.

For the FH-binding inhibition assay, FH in serum were bound by ExPEC as well as magnetic beads-coupled recombinant protein. The value of OD_450_ indicated the level of FH recruited by bacteria. The results indicated that the co-incubation of magnetic beads-coupled GST-fusion proteins AckA, FbaA, FrdA, LDH, LpdA, Pdh, and PpsA with ExPEC significantly decreased the bacterial FH-binding ability compared Pgk and GST tag (*p* < 0.05, **Figure 3C**).

These results demonstrated that recombinant Pdh, AckA, FbaA, FrdA, LDH, LpdA, and PpsA proteins possessed FH-binding ability whereas recombinant Pgk protein did not. In addition, the ELISA plate binding assay indicated that these seven recombinant proteins interacted with FH in a dose-dependent manner. The FH-binding inhibition assay suggested that these seven proteins played a role in FH recruitment of ExPEC.

### Localization of CMEs on ExPEC Outer Membrane

The outer membrane localization is an important precondition for the interaction between ExPEC and FH. Proteins extracted by Triton X-114 were hydrophobic proteins, which contained outer membrane proteins as well as inner membrane proteins. Therefore, it was necessary to detect the membrane location of these seven CMEs. Polyclonal antibodies against the seven CMEs and the OmpA were prepared. To examine the specificity of these antibodies, Western blotting was performed. Single positive bands were observed, indicating that the antibodies were specific to these recombinant proteins (**Figure 2**).

**Figure 4:**
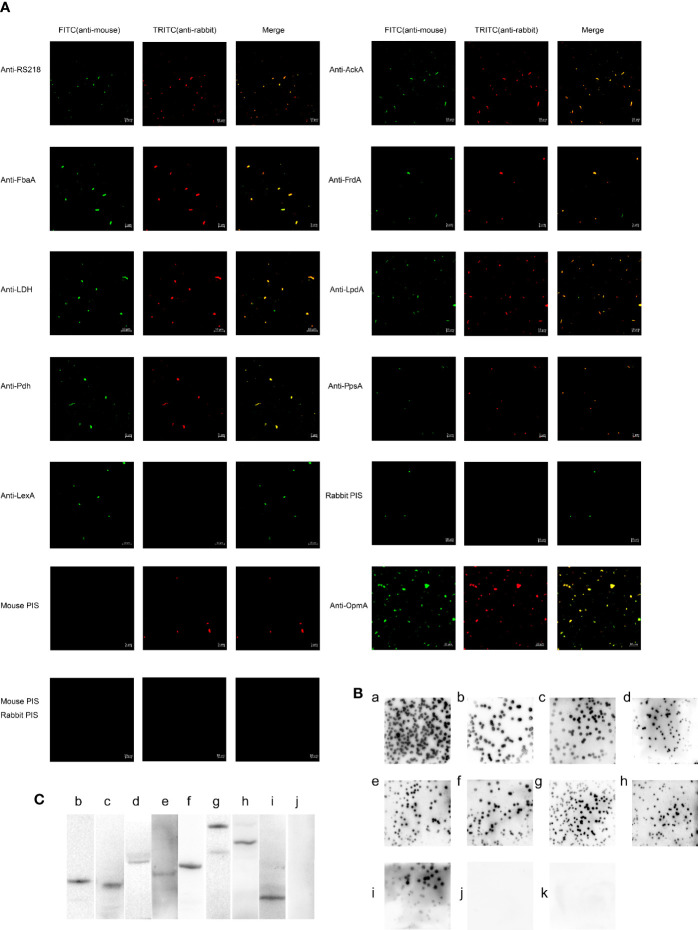
The outer membrane localization of CMEs on ExPEC. **(A)** Immunofluorescence of ExPEC strain treated with the mixture of mouse anti-RS218 anti-serum and rabbit anti-RS218 anti-serum (positive control) or rabbit anti-OmpA antibody (out membrane protein control) or rabbit anti-CME antibody or rabbit anti-LexA antibody. The mixture of mouse anti-RS218 anti-serum and rabbit PIS, the mixture of rabbit anti-RS218 anti-serum and mouse PIS, and the mixture of mouse PIS and rabbit PIS was used as negative controls. Detection was conducted by incubating with FITC-conjugated anti-mouse IgG and TRITC-conjugated anti-rabbit IgG. **(B)** Immunoblotting reaction of ExPEC strain RS218 colonies. Colonies were covered with NC membrane followed by incubation with rabbit anti-RS218 anti-serum (a), anti-CMEs antibodies (b, anti-AckA; c, anti-FbaA; d, anti-FrdA; e, anti-LDH; f, anti-LpdA; g, anti-Pdh; and h, anti-PpsA), anti-OmpA antibody (i); anti-LexA antibody (j), and rabbit PIS (k). **(C)** The outer membrane proteins were extracted and detected by Western blotting (b, AckA; c, FbaA; d, FrdA; e, LDH; f, LpdA; g, Pdh; h, PpsA, i, OmpA; j, LexA).

To detect the outer membrane location of AckA, FbaA, FrdA, LDH, LpdA, Pdh, and PpsA on ExPEC, immunofluorescence assays were performed. **Figure 4A** shows that strong signals were obtained when bacteria treated with anti-serum against RS218 and antibody against OmpA, AckA, FbaA, FrdA, LDH, LpdA, Pdh, and PpsA, whereas bacteria treated with PIS and LexA antibody remained negative. Experiments using PIS remained negative in all approaches, suggesting the limited occurrence of cross-reactions. The LexA protein of *E. coli* is a transcriptional repressor located in cytoplasm. The absence of reactivity of the LexA antibody indicated that the detection of cytosolic proteins was excluded in this assay.

In the colony blotting experiments, the anti-RS218 anti-serum, anti-OmpA antibody, and anti-CMEs antibodies reacted with the immobilized proteins from the outer membrane of bacteria colonies, whereas no reactivity of PIS was observed (**Figure 4B**). The absence of reactivity of anti-LexA antibody suggested that LexA was not detected from the outer membrane of the bacteria colonies.

In the Western blotting, the outer membrane proteins were detected using OmpA, CMEs or LexA antibodies and HRP-conjugated anti-rabbit IgG. The positive band (**Figures 4Cb–h**) suggested that these CMEs were outer membrane proteins as compared with positive control (**Figures 4Ci**). The negative result of LexA indicated that the extracted outer membrane protein was not contaminated by cytoplasmic protein (**Figure 4Cj**).

The above results determined that AckA, FbaA, FrdA, LDH, LpdA, Pdh, and PpsA were located on the outer membrane of ExPEC.

### ExPEC Binding to FH Significantly Decreased C3b Deposition

**Figure 5:**
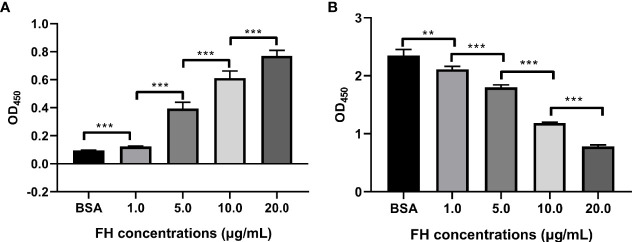
FH recruitment decreased the C3b deposition on ExPEC. Bacteria pre-incubated with purified human FH (1.0, 5.0, 10, or 20 μg/ml) or BSA, and incubated with 10% FH-depleted human serum. The FH recruitment levels **(A)** and the C3b deposition levels **(B)** were detected by ELISA. The data are expressed as the mean ± SEM of three independent experiments. The statistical significance of differences between each pair was determined using the unpaired *t* test (**p < 0.01; ***p < 0.001; ns, no significance).

The detection of FH-depleted serum by Western blotting revealed that 10% FH-depleted serum did not contain FH (**Supplementary Figure S1**). The ELISA assay was performed to investigate whether ExPEC binding to FH contributes to the decrease in C3b deposition. **Figure 5A** shows that ExPEC recruited pure FH in a dose-dependent manner (*p* < 0.05). The C3b deposition was significantly decreased when bacteria pre-incubated with FH (*p* < 0.05, **Figure 5B**). In addition, as the level of FH recruitment increased significantly, the level of C3b deposition decreased significantly (*p* < 0.05, **Figures 5A, B** **)**. These results also suggested that the FH recruitment reduced the C3b deposition on ExPEC.

### Binding to FH Significantly Increased Anti-Opsonophagocytosis of ExPEC

**Figure 6:**
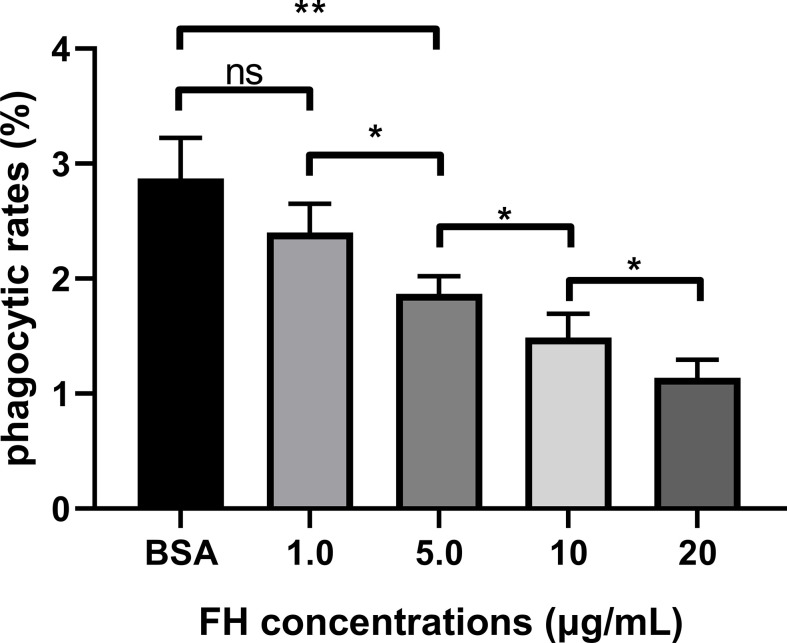
FH recruitment contributed to the anti-opsonophagocytic ability of ExPEC. ExPEC strain RS218 pre-incubated with purified human FH and incubated with 10% FH-depleted human serum. Then the opsonized bacteria were infected to THP-1 cells or performed plate counting. The phagocytic rates were determined as ratios of the number of bacteria recovered from THP-1 to those recovered from human serum. The data are expressed as the mean ± SEM of three independent experiments. The statistical significance of differences between each pair was determined using the unpaired *t*-test (*p < 0.05; **p < 0.01; ns, no significance.).

The opsonophagocytosis assay was performed to determine the effect of FH recruitment on the anti-opsonophagocytosis of ExPEC. The phagocytosis rates of the pre-incubation FH group (5.0, 10, and 20 μg/ml, respectively) were significantly lower than that of the BSA pre-incubation group and 1.0 μg/ml FH pre-incubation group (*p* < 0.01, **Figure 6**). In addition, the phagocytosis rates significantly decreased with the two-fold increase in FH pre-incubation concentration. However, pre-incubating with 1.0 μg/ml of FH had no significant effect on the anti-opsonophagocytosis capacity of the ExPEC (*p* > 0.05). These results showed that pre-incubated with a sufficient concentration of FH significantly contributed to the anti-opsonophagocytic ability of ExPEC in an FH-concentration-dependent manner.

## Discussion

The complement system plays a role in identifying pathogens in the bloodstream (Putrins et al., 2015). Bacteria have developed effective strategies to avoid the killing effect mediated by the complement system. Some bacteria subvert complement attack by recruiting complement regulators, which results in diminished bacterial lysis or weakened opsonophagocytosis (Kraiczy and Würzner, 2006). For example, the outer membrane protein Rck of *Salmonella enterica* binds to FH to reduce the deposition of C3b and MACs, which protects bacteria from complement-mediated killing (Ho et al., 2010). Secreting proteases to degrade crucial complement components is another evasion strategy used by bacteria. For example, elastase and alkaline protease of *P. aeruginosa* degrade key recognition molecules of the complement system, namely, C1q and C3, aborting complement-mediated bacterial killing (Hong and Ghebrehiwet, 1992). Some bacteria express complement inhibitors. For example, *Borrelia burgdorferi* express the CD59-like protein to inhibit MACs formation (Lambris et al., 2008). In addition, the cell wall of Gram-positive bacteria such as *S. pneumoniae* protects bacteria against the lysis caused by MACs (Joiner et al., 1983).

It has been reported that *E. coli* utilizes some strategies to binding FH. The polysialic acid, the main component of capsule, protect the bacteria from complement attack by facilitating the binding of FH (Ram et al., 1998; McCarthy et al., 2018). The lipid A, a toxic component of LPS, was competitively bound by FH and C1q (Tan et al., 2011). The outer membrane protein W (OmpW) of *E. coli* K12 strain K99+ bound to FH to protect bacteria against complement attack (Li et al., 2016). ExPEC such as K1 RS218 cause bloodstream infections, while the non-pathogenic *E. coli* such as K12 MG1655 may not be capable to survive in the blood. Although the capsule binds FH, it also covers the bacterial surface proteins to some extent, hindering interactions between bacteria and host factors (Xu et al., 2016). In addition, the LPS and OmpW existed in both pathogenic *E. coli* and non-pathogenic *E. coli*, which indicated that they were not sufficient to resist complement attack. There may be additional virulence factors in ExPEC that promoted its survival in bloodstream. Evasion of complement attack is one of the important mechanisms for bacteria to survive in the blood (Blom et al., 2009). Recruiting FH to prevent C3b deposition is an effective strategy for bacteria to resist complement attack (Kraiczy and Würzner, 2006). *E. coli* that cause bloodstream infections may have additional FH-binding proteins. Exploring these FH-binding proteins is one of the purposes of this study.

By desthiobiotin pull-down and LC-MS/MS analysis, we identified ExPEC membrane proteins with FH-binding function. In this study, some CMEs, including glycolysis enzymes, pyruvate metabolic enzymes, and tricarboxylic acid (TCA) cycle enzymes, were demonstrated as novel FH-binding proteins such as AckA, FbaA, Frda, LDH, LpdA, Pdh, and PpsA. Generally, CMEs are involved in metabolic processes. However, some glycolytic enzymes have been regarded as moonlighting proteins in many bacteria (Henderson and Martin, 2011). Moonlighting protein refers to a protein that exhibits more than one function (Henderson and Martin, 2011). There are many highly conserved proteins such as metabolic enzymes and molecular chaperones that have been identified as moonlighting proteins. FbaA, which is involved in glycolysis and pentose phosphate pathway, catalyzing the cleavage of fructose 1,6-diphosphate to dihydroxyacetone phosphate and glyceraldehyde 3-phosphate, is a moonlighting protein with FH-binding and plasminogen-binding functions in *S. pneumoniae* and *N. Meningitidis* (Blau et al., 2007; Tunio et al., 2010; Li et al., 2017). Pdh, which is involved in pyruvate metabolism, TCA cycle, and glycolysis, catalyzing pyruvic acid to acetyl-CoA, is a moonlighting protein in *Mycoplasma pneumoniae* that binds to human extracellular matrix such as fibronectin, lactoferrin, and vitronectin (Dallo et al., 2002; Gründel et al., 2016). LDH, a glycolysis and pyruvate metabolic enzyme, catalyzing the conversion of lactate to pyruvate, acts as a moonlighting protein, which binds to transferrin in *Mycobacterium tuberculosis* and to human extracellular matrix in *M. pneumoniae* (Boradia et al., 2014; Gründel et al., 2016). In addition, another four CMEs, namely, AckA, LpdA, FrdA, and PpsA, were randomly selected to study their FH-binding function. AckA is involved in pyruvate metabolism, catalyzing the formation of acetyl phosphate from acetate and ATP. LpdA is the pyruvate dehydrogenase E3 subunit that is involved in glycolysis, the TCA cycle, and pyruvate metabolism. FrdA is involved in the TCA cycle and pyruvate metabolism. PpsA is involved in pyruvate metabolism.

Carbohydrate metabolism is an essential metabolic process of bacteria, which not only provides energy for cell metabolism, but also provides building blocks for biomolecular synthesis (Durica-Mitic et al., 2018). As these seven CMEs participate in the carbohydrate metabolism, the deletion of their coding genes may have potential impacts on the global metabolism and even biomolecular synthesis of bacteria. Furthermore, the *fbaA* was determined to be one of essential genes for *E. coli*, which cannot be knockout (Goodall et al., 2018; McCarthy et al., 2018). Therefore, gene knockout strains of CMEs were not constructed in this study. The FH binding inhibition assay of magnetic beads coupled with recombinant protein were utilized to explore the effects of these seven CMEs on FH recruitment. The result suggested that these seven CMEs played a role in FH recruitment on ExPEC.

Generally, metabolic enzymes are cytosol-located proteins. However, it has been reported that bacterial glycolytic enzymes, such as FbaA, Pdh, and LDH, are outer membrane-located moonlighting proteins (Blau et al., 2007; Gründel et al., 2016). Results of immunofluorescence, colony blotting assays, and Western blotting targeting outer membrane proteins confirmed that seven CMEs were outer membrane proteins of ExPEC strain RS218. Besides, these seven CMEs were not detected in the outer membrane of the *E. coli* K12 MG1655 (**Supplementary Figure S2**). The lack of moonlighting protein CMEs on the outer membrane may be one of the reasons why K12 is a non-pathogenic *E. coli*.

The bioinformatics predication showed that these seven CMEs mentioned above have neither signal peptide nor transmembrane region (**Table 3**). The soluble expression of these seven moonlighting proteins also suggested the lack of transmembrane regions. Some studies have reported that cytoplasmic proteins with moonlighting function were released through bacterial lysis or through leakage during bacterial division or caused by membrane-damaging activity. For example, the glyceraldehyde 3-phosphate dehydrogenases (GAPDH) of Group B streptococcus was reported as a surface protein released upon cell lysis (Oliveira et al., 2012). The FbaA of *Staphylococcus aureus* has been determined that was released out of bacteria either by leakage during cell division or leakage induced by α-type phenol-soluble modulins induced membrane destruction (Ebner et al., 2016; Ebner et al., 2017). Other studies have been reported that the type III secretion system (T3SS) and type IV secretion system (T4SS) of bacteria were responsible for the translocation of cytoplasmic proteins. For example, the GAPDH of enteropathogenic *E. coli* secreted to bacterial surface through the T3SS and the alcohol dehydrogenase of *Coxiella burnetii* was secreted through the T4SS (Samoilis et al., 2010; Aguilera et al., 2012). It has been implied that the hydrophobic regions (hydrophobic amino acids, motifs, or domains) existed in the structure of secreted cytoplasmic proteins, or were exposed after post-translational proteolysis and conformational changes, enabling cytoplasmic proteins to perform membrane-anchoring functions (Widjaja et al., 2017; Jeffery C. J., 2018b). For example, the EF-Tu of *S. aureus* was identified to have different size of cleavage fragments on the bacterial surface (Widjaja et al., 2017). The CMEs antibodies used in this study were against a certain peptide of CMEs. Therefore, it is difficult to confirm whether these CMEs undergo post-translational proteolysis, as the antibody cannot detect all segments of the amino acid sequence of the protein. Mechanisms of these CMEs secreting and anchoring to the outer membrane still require further research.

Phagocytosis is a critical event of the innate immune response against pathogenic bacteria (Lim et al., 2017). Phagocytosis can be facilitated by serum opsonin such as C3b (Agrahari et al., 2016). When C3b is deposited on pathogens, it can be recognized by phagocytic cells, triggering opsonophagocytosis (Blom et al., 2009; Bajic et al., 2013). It has been reported that the recruitment of FH by Gram-positive bacteria leads to the reduction of C3b deposition as well as the attenuation of opsonophagocytosis (Haapasalo et al., 2008; Melin et al., 2009). However, studies on opsonophagocytosis in Gram-negative bacteria are limited. The FH recruitment level and C3b deposition level of ExPEC after incubation with FH and FH-depleted serum were assessed. The results suggested that FH recruitment contributed to inhibiting C3b deposition on ExPEC in an FH-concentration-dependent way (**Figures 5A, B** **)**. The incubation of ExPEC with FH-depleted serum induced a significant increased C3b deposition, suggesting that FH plays an important role in the complement resistance of ExPEC. Furthermore, we found Gram-negative bacteria ExPEC binding to FH significantly enhanced the resistance to opsonophagocytosis in an FH-concentration-dependent manner (**Figure 6**). These results provide new insights for Gram-negative bacteria escaping opsonophagocytosis.

This study screened the FH-binding membrane proteins of ExPEC. The dose-dependent FH-binding function of seven CMEs was demonstrated. In addition, co-incubation of magnetic beads coupled with seven recombinant CMEs with ExPEC in human serum significantly reduced the binding of FH to bacteria, suggesting that these seven CMEs played a role in FH recruitment on ExPEC. We have also confirmed the outer membrane location of these CMEs. Screening and identification of FH-binding proteins in ExPEC contributes to elucidate the molecular mechanism of ExPEC binding to FH. In addition, our study has demonstrated that ExPEC binding to FH significantly reduced the C3b deposition on bacteria as well as the opsonophagocytosis of THP-1 in an FH-dose-dependent manner. These findings provide avenues to further evaluate the molecular mechanisms of ExPEC complement escape.

## Data Availability Statement

The datasets presented in this study can be found in online repositories. The names of the repository/repositories and accession number(s) can be found below: http://www.proteomexchange.org/, PXD020521.

## Author Contributions

JD, YS, and BX conceived of the study. YS, QG, XW, and RC performed the experiments. YS, XZ, FT, and FX analyzed experimental results. YS and BX wrote the manuscript. All authors contributed to the article and approved the submitted version.

## Funding

This work was supported by the National Natural Science Foundation of China (grant no. 31872479 and 31702252) and Fund of Priority Academic Program Development of Jiangsu Higher Education Institutions (PAPD). The funders had no role in study design, data collection, and interpretation, or the decision to submit the work for publication.

## Conflict of Interest

The authors declare that the research was conducted in the absence of any commercial or financial relationships that could be construed as a potential conflict of interest.
