# Supplementary material for: Factor H Is Bound by Outer Membrane-Displayed Carbohydrate Metabolism Enzymes of Extraintestinal Pathogenic Escherichia coli and Contributes to Opsonophagocytosis Resistance in Bacteria
Source: Front Cell Infect Microbiol. 2021 Jan 25;10:592906. doi: 10.3389/fcimb.2020.592906 (PMC7868385; doi:10.3389/fcimb.2020.592906)
Supplement: Supplementary file 1 [file Table_1.docx]

Supplement Table S1 Peptides used for antibody preparation.

| Proteins | Sequence of peptides |
| --- | --- |
| AckA | TSDCRYVEDNYATK |
| FbaA | EEDGVDNSHMDAS |
| FrdA | CTERDDVNFLKHT |
| LDH | RILKNMSDLSLETT |
| LpdA | EGSITDLPNPKAK |
| Pdh | SERFPNDVDPIET |
| PpsA  OmpA | DRDSGVVSELFDERN  LFTFNKATLKPEGQ |
