# Supplementary figures and images for: Factor H Is Bound by Outer Membrane-Displayed Carbohydrate Metabolism Enzymes of Extraintestinal Pathogenic Escherichia coli and Contributes to Opsonophagocytosis Resistance in Bacteria
Source: Front Cell Infect Microbiol. 2021 Jan 25;10:592906. doi: 10.3389/fcimb.2020.592906 (PMC7868385; doi:10.3389/fcimb.2020.592906)

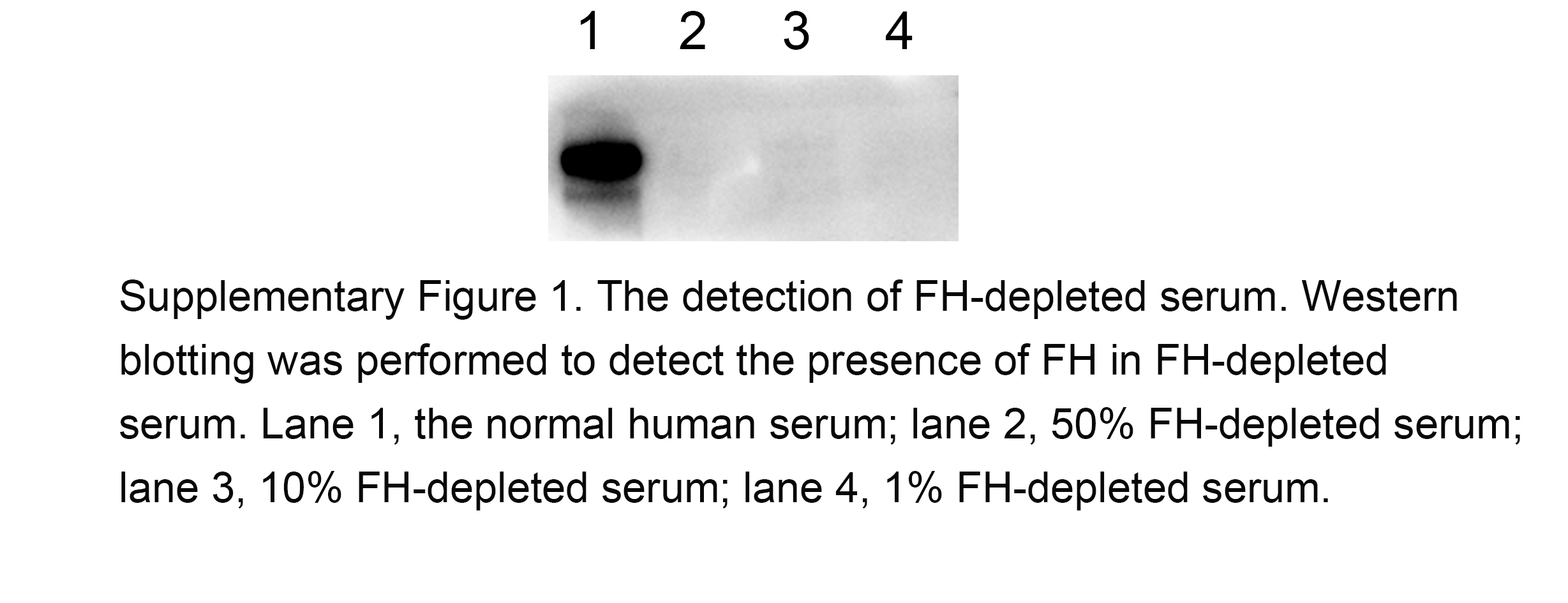

Supplement: Supplementary file 3 [file Image_1.tif]

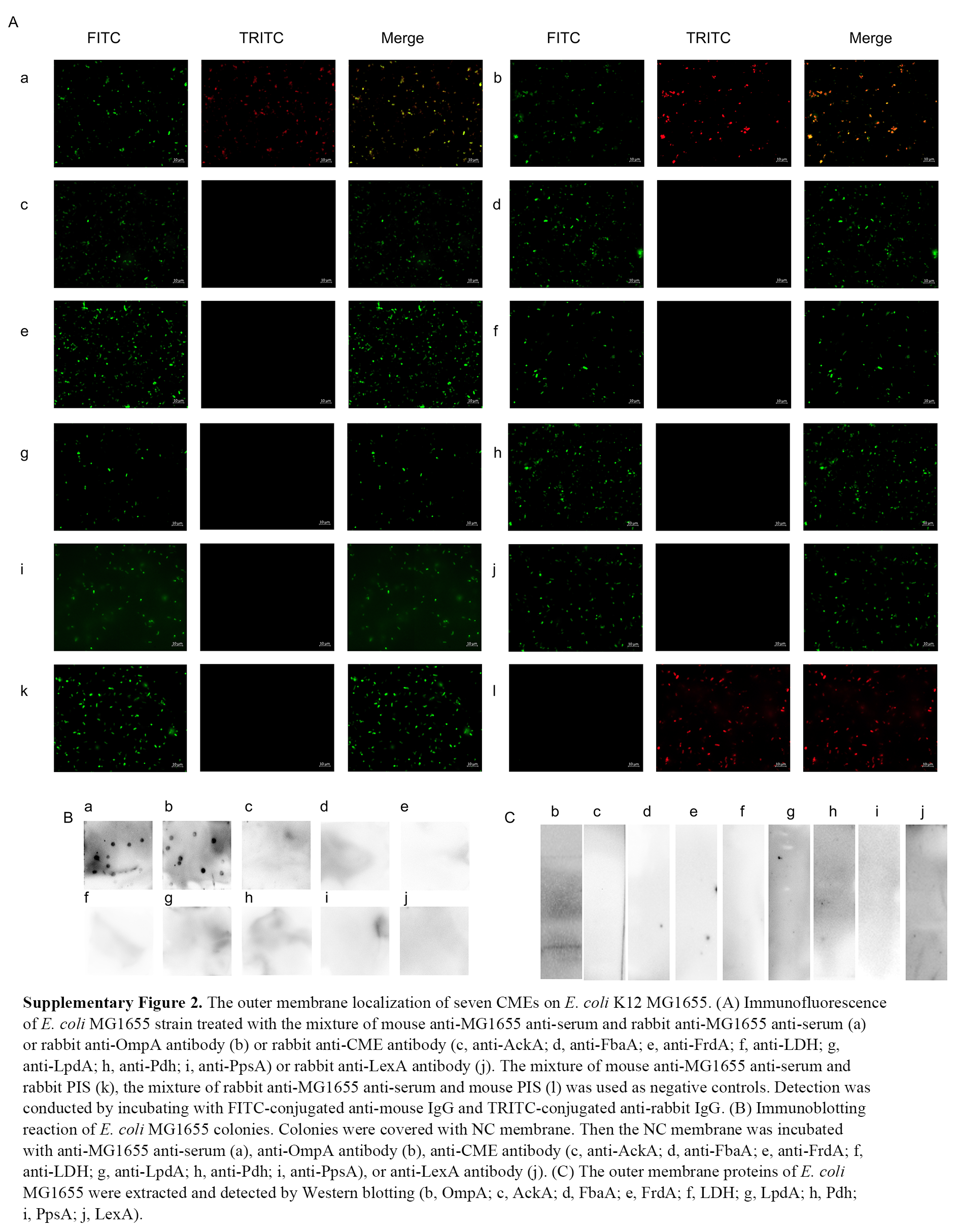

Supplement: Supplementary file 4 [file Image_2.tif]
